# Enhanced laboratory surveillance of respiratory infection disclosed the rapid rise of enterovirus D68 cases, northern Italy, August to September 2024

**DOI:** 10.2807/1560-7917.ES.2024.29.41.2400645

**Published:** 2024-10-10

**Authors:** Elena Pariani, Antonio Piralla, Laura Pellegrinelli, Federica Giardina, Vincenzo Navobi Porrello, Greta Romano, Cristina Galli, Laura Sandri, Guglielmo Ferrari, Sandro Binda, Luigi Vezzosi, Gabriele Del Castillo, Sabrina Buoro, Danilo Cereda, Fausto Baldanti, Respiratory viruses pandemic preparedness group Lombardy, Respiratory viruses pandemic preparedness group Lombardy, Francesca Rovida, Antonino Pitrolo, Sara Uceda Renteria, Annapaola Callegaro, Marco Arosio, Claudio Farina, Diana Fanti, Alice Nava, Federica Novazzi, Nicasio Mancini, Alessandro Mancon, Valeria Micheli, Fabio Sagradi, Sergio Malandrin, Annalisa Cavallero, Olivia Turri, Daniela Campisi, Maria Fasano, Maddalena Soncini, Maria Grazia Marin, Bianca Osnaghi, Massimo Oggioni, Beatrice Pini, Virginia Federico, Francesco Scovenna, Federica Morani, Michela Viscardi, Manuel Maffeo, Lucia Crottogini

**Affiliations:** 1 Department of biomedical sciences for health, University of Milan, Milan, Italy; 2 Microbiology and virology department, Fondazione IRCCS Policlinico San Matteo, Pavia, Italy; 3 Department of clinical, surgical, diagnostic and paediatric sciences, University of Pavia, Pavia, Italy; 4 Department of public health, experimental and forensic medicine, University of Pavia, Pavia, Italy; 5 Directorate General for Health, Lombardy Region, Milan, Italy; 6 The members of the Respiratory viruses pandemic preparedness group Lombardy are listed under Collaborators

**Keywords:** Enterovirus, Enterovirus D68, respiratory infections, laboratory surveillance, influenza-like illness surveillance

## Abstract

We report a considerable increase in enterovirus D68 (EV-D68) cases since July 2024, culminating in an ongoing outbreak of acute respiratory infections in northern Italy, accounting for nearly 90% of all enterovirus infections. The outbreak was identified by community- and hospital-based surveillance systems, detecting EV-D68 in individuals with mild-to-severe respiratory infections. These strains belonged to B3 and a divergent A2 lineage. An increase in adult cases was observed. Enhanced surveillance and molecular characterisation of EV-D68 across Europe are needed.

The emergence of enterovirus D68 (EV-D68) as a notable respiratory pathogen with the potential to cause paralysis similar to poliomyelitis and called acute flaccid myelitis (AFM) represents an important public health concern [1]. Here we describe an ongoing increase in EV-D68 cases in Lombardy, northern Italy, detected by the surveillance system of respiratory infections.

## Surveillance of enterovirus D68

No dedicated surveillance systems have been established in Europe to monitor EV-D68 [2,3]. In response to this, and as part of a broader surveillance of respiratory infections, a laboratory-based surveillance network for respiratory infections was broadened in the Lombardy region of northern Italy (population nearly 10 million), in September 2023, by including emergency departments (ED) into the surveillance. The network comprises two distinct surveillance systems for respiratory viruses. One is a community-based system, operated by a public health laboratory that serves as the regional reference laboratory for Lombardy within the Italian respiratory virus surveillance network (RespiVirNet) (https://www.iss.it/en/respivirnet) and is primarily engaged in the testing of the community samples for influenza-like illness (ILI). The other is a hospital-based system, which encompasses microbiological diagnostic laboratories that are focused on the testing of patients, particularly those visiting the ED or being admitted to hospital in the event of a severe acute respiratory infection (SARI).

## Detection and characterisation of enterovirus D68

Molecular detection of enteroviruses is currently conducted on a routine basis in respiratory samples collected from ILI and ED/SARI cases. All samples identified as positive for enterovirus by molecular tests carried out by each laboratory involved in the network are immediately typed, which is conducted by two reference laboratories (Fondazione IRCCS Policlinico San Matteo and University of Milan) using a direct EV-D68 real-time PCR assay [4,5] and Sanger sequencing of the VP1/VP3 or VP2/VP4 regions of the virus [6,7]. Whole genome sequencing (WGS) is conducted for a subset of EV-D68 strains from both community ILI and ED/SARI cases, from patients of varying ages, over different weeks and according to viral load (cycle threshold < 30) to evaluate the phylogenetic relationships. In detail, EV-D68 WGS is performed with three overlapping amplicons [8]. Genomic libraries are prepared using the Nextera XT Library Preparation Kit (Illumina, San Diego, the United States (US)), and sequencing is performed on the MiSeqDx platform using the MiSeq Reagent kit V2 (Illumina). The resulting sequences are analysed using INSaFLU (https://insaflu.insa.pt/), a user-friendly bioinformatics web-based tool designed for the analysis of primary sequencing data [9].

In order to monitor enterovirus activity, the weekly positivity rate is tracked through community and ED surveillance. The positivity rate is expressed as the number of enterovirus-positive samples of the total number of respiratory samples collected from patients presenting with ILI (community surveillance) or SARI (ED surveillance).

## Enterovirus D68 surge

### Influenza-like illness community surveillance programme

**Figure 1:**
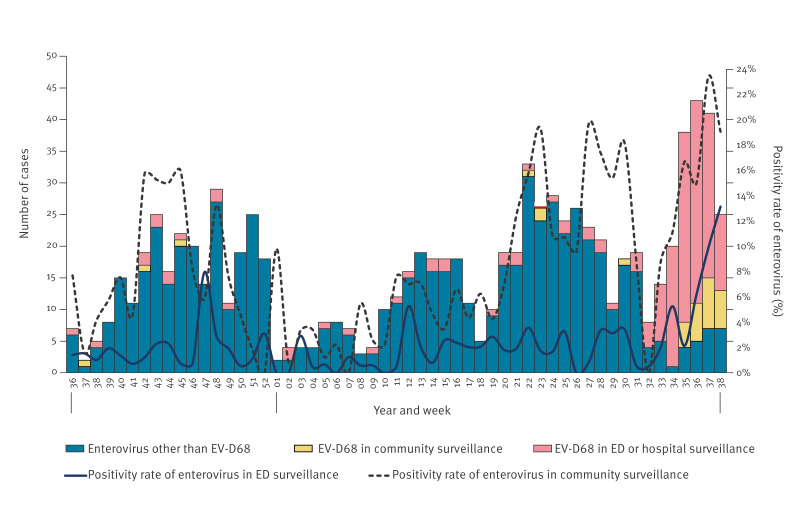
Epidemic curve of enterovirus and enterovirus D68 cases in Lombardy, northern Italy, 4 September 2023 (week 36)–22 September 2024 (week 38) (n = 952)

ED: emergency department; EV-D68: enterovirus D68.

A total of 2,478 respiratory specimens from ILI outpatients were examined between 1 January and 22 September 2024 as part of the ILI community surveillance programme. The enterovirus genome was identified in 7.7% (n = 192) of the specimens analysed. The presence of enterovirus was identified from week 1 2024 onwards, with the weekly enterovirus-positivity rate exceeding 10% for the first time (12.5%; 10/80) in week 21 2024 (end of May) and reaching a peak of 23.4% (11/47) in week 37 2024 (beginning of September) (Figure 1). Of the 192 respiratory specimens that tested positive for enterovirus, 28 (14.6%) were EV-D68, with four of five samples positive in week 35 2024 (end of August) and all six samples positive in week 36 2024 (beginning of September).

The weekly enterovirus positivity rate is expressed as the number of enterovirus-positive samples of the total number of respiratory samples collected from patients with influenza-like illness in the community surveillance or in the emergency department surveillance.

### Severe acute respiratory infection in emergency departments and admitted patients

In the ED setting, the mean of the weekly positivity rate of enterovirus was 2.1%, varying between 0.0% in week 1 2024 (beginning of January) and 13.1% (29/221) in week 38 2024 (mid-September) (Figure 1). Between 1 January and 22 September 2024, all 461 respiratory samples positive for enterovirus from ED/SARI inpatients were further characterised, and EV-D68 was identified in 34.9% (n = 161) of these samples.

Most of the EV-D68 positive samples (161/189; 85.2%) were obtained from surveillance of patients visiting ED or hospitalised for SARI, and the remaining 14.8% (28/189) from ILI patients. Overall, the data indicate that EV-D68 exhibited a low intensity of circulation up to week 29 2024 (mid-July), with an overall EV-D68-positivity rate of 6.8% and a mean weekly positivity rate of 4.5% (range: 0–25%). Subsequently, a statistically significant (Chi-square test based on binomial distribution) increase was observed, reaching 62.7% (range: 5.6–95.0%) as of 22 September 2024. From week 34 2024 (mid-August) onwards, EV-D68 constituted 86.2% (131/152) of cases positive for enterovirus. Despite the high increase in EV-D68 circulation, no cases of acute flaccid paralysis (AFP) or AFM were identified that were related to EV-D68 infection in the population, according to the results of the AFP surveillance conducted in Lombardy by the public health laboratory (University of Milan).

### Age profile of enterovirus D68 cases

The median age of the EV-D68 cases was 17.2 years (interquartile range (IQR): 59 years; range: 0–98 years). The community ILI cases with EV-D68 (median age: 22 years; IQR: 41 years) tended to be older than the ED/SARI cases with EV-D68 (median age: 14.7 years; IQR: 61; p = 0.34).

Among the community ILI cases with EV-D68, the median age was significantly higher (p = 0.001) compared with the median age of ILI cases infected with other enteroviruses (3 years; IQR: 7 years). Among the ED/SARI cases, the median age of cases with other enteroviruses was 2 years (IQR: 5 years).

### Enterovirus sequencing

Information on enterovirus types other than EV-D68 was obtained by sequencing selected EV-D68-negative samples identified in 2024. These data were available for 158 of 238 (66.4%) strains (82 from community ILI and 76 from ED/SARI). Twenty different enterovirus genotypes were identified: the most common were coxsackievirus B3/B4 (ILI: n = 24; 29.3% and ED/SARI: n = 23; 30.3%) and echovirus 6 (ILI: n = 11; 30.4% and ED/SARI: n = 12; 15.8%).

**Figure 2:**
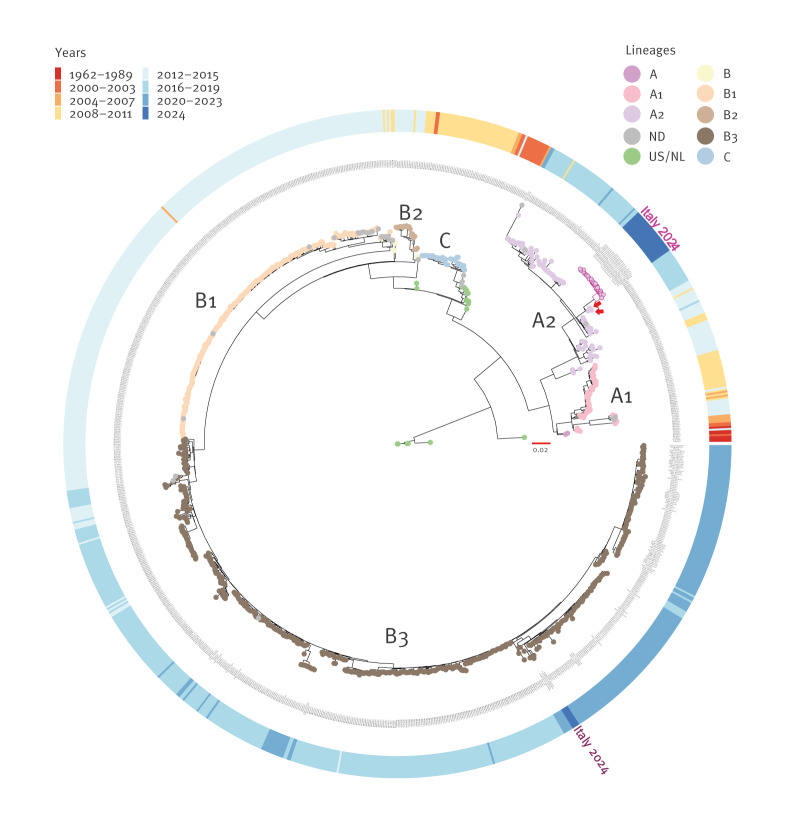
Maximum likelihood phylogenetic tree of complete coding sequence regions of enterovirus D68, 1962–2024 (n = 1,057)

We whole genome sequenced 29 EV-D68 strains (GenBank accession numbers: PQ426627-PQ426654): four strains belonged to the B3 lineage and 25 to a divergent A2 lineage (Figure 2, highlighted in purple) and compared with data from 1,028 sequences downloaded from the National Center for Biotechnology (NCBI) GenBank database (https://www.ncbi.nlm.nih.gov/genbank/). The nearest EV-D68 strains in the A2 lineage were from patients in the Netherlands (MN726801.1) and China (MW697455.1) in 2019 and in France (MK105986.1) in 2018 (Figure 2, red arrow). We further compared the Italian strains belonging to the A2 lineage with MN726801.1, MW697455.1 and MW697455.1 by pairwise distance. The average nucleotide identity between the Italian and the other strains was 96.9% (range: 96.6–97.5%).

ND: not determined.

The EV-D68 strains (n = 29) identified in this study are highlighted in purple. The outer layer represents the sequencing years (1962–2024), and the nodes of the tree are coloured based on the lineage cluster. The alignment was performed with MAFFT v7.525 [14] and the maximum likelihood phylogenetic tree was constructed using IQ-TREE multicore version 2.3.3 [15] under the GTR+G+I nucleotide substitution model chosen according to BIC score (Bayesian Information Criterion) as it was the best-fitting one selected by ModelFinder. The robustness of branches was evaluated using the Shimodaira–Hasegawa approximate likelihood-ratio test (SH-aLRT) and UltraFast bootstrap approximation tests.

**Figure 3:**
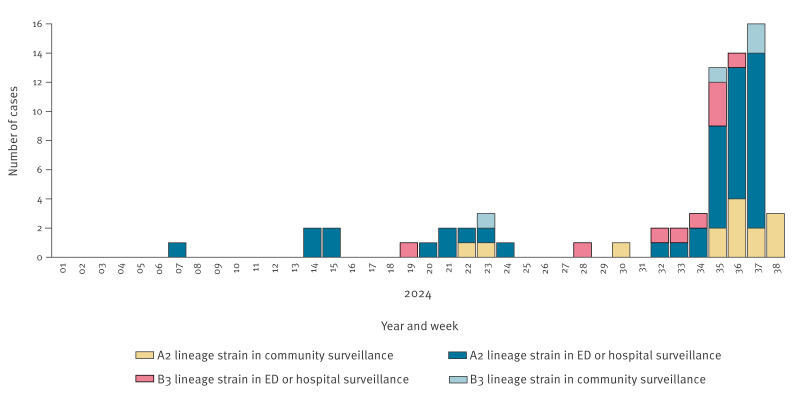
Distribution of enterovirus D68 lineages in Lombardy, northern Italy, 1 January 2024 (week 1)–22 September 2024 (week 38) (n = 70)

ED: emergency department; ILI: influenza-like illness; NGS: next generation sequencing; SARI: severe acute respiratory infection.

Of sequencing by Sanger and NGS, a sequence was obtained for 37.0% (70/189) of all EV-D68 cases. Overall, 81.4% (57/70) were categorised as A2 lineage, while 18.6% (13/70) as B3 lineage. The median age of cases with EV-D68 A2 lineage was higher than that of cases with B3 lineage (44.3 years vs 4.6 years; p < 0.001). Figure 3 shows the distribution of EV-D68 lineages.

Data from 70 sequences obtained either by Sanger or NGS from ILI community surveillance (n = 18) and from ED/SARI surveillance (n = 52).

## Discussion

The EV-D68 typically circulates during late summer and autumn, with a peak August–October in temperate regions. Before the COVID-19 pandemic, historical data indicated the occurrence of biannual cycles of outbreaks [10,11]. However, the pandemic period was characterised by a notable absence of such cycles, which was attributed to the implementation of public health and social measures (PHSM). Currently, there is evidence suggesting that EV-D68 is circulating annually, with variations in the timing and intensity of outbreaks observed from year to year [12]. Outbreaks can be sporadic, with certain years showing more notable increases in cases, especially among children [13]. However, the shift in the age profile, coupled with the high incidence of EV-D68, particularly in inpatients, indicates that the virus predominantly affects persons aged > 15 years, thereby demonstrating a change in the epidemiological characteristics of EV-D68.

It remains to be seen whether this change is due to EV-D68 genome mutation and evolution, a different level of population immunity or some other unforeseen characteristics. It is crucial to acknowledge that PHSM implemented during the pandemic period disrupted the previous typical viral transmission, reduced exposure to respiratory viruses and altered population immunity. These factors are likely to have contributed to a shift in the circulation patterns of respiratory viruses, including EV-D68 [12].

Whatever the cause, the outbreak has highlighted the need for a public health response. The implementation of a laboratory-based surveillance programme, in collaboration with public health and clinical laboratories, has enabled the real-time capture of a new upsurge of EV-D68, which causes mild-to-severe respiratory infections, primarily affecting adults rather than young children [13]. The incorporation of EV-D68 surveillance into comprehensive respiratory surveillance systems has enabled the measurement of the incidence of EV-D68 at the community level. This approach provides valuable data on virus circulation and enables the timely identification of potential cases of severe illness. In particular, the inclusion of EDs in the respiratory infection surveillance system is part of the pandemic preparedness plans of the region. This comprehensive respiratory infection surveillance system is of critical importance for the timely identification and response to potential outbreaks, enabling prompt alerting of the public health authorities and the subsequent modification of public health practices. The annual cost to the Lombardy region of this surveillance is ca 2.4 million euros.

The findings from this surveillance network highlight the critical importance of enhancing laboratory-based surveillance efforts and improving rapid diagnostic capabilities for EV-D68. Such improvements are essential for guiding effective public health interventions, ensuring early detection of outbreaks and mitigating the impact of the virus on public health globally. The incorporation of EV-D68 surveillance into respiratory infection surveillance as part of a pandemic preparedness plan enabled us to ascertain the incidence of EV-D68 at the population level and to identify potential cases of severe illness in a timely manner. Furthermore, whole genome sequencing of enteroviruses is vital for the assessment of emerging EV-D68 variants.

## Conclusion

The network findings emphasise the necessity for enhanced laboratory-based surveillance and rapid diagnostic capabilities for EV-D68 at both national and international levels to inform public health interventions.
